# Symptoms of Depression and Anxiety among Women attending Primary Health Care in Gilgit-Baltistan (GB), Pakistan

**DOI:** 10.12669/pjms.336.13736

**Published:** 2017

**Authors:** Sadiq Hussain, Sabih Ahmad, Anum Zahra, Naila Jabeen

**Affiliations:** 1 Sadiq Hussain, Department of Behavioral Sciences, Karakoram International University Gilgit, Pakistan; 2 Sabih Ahmad, Department of Psychiatry, Combined Military Hospital, Gilgit, Pakistan; 3 Anum Zahra, Department of Behavioral Sciences, Karakoram International University Gilgit, Pakistan; 4 Naila Jabeen, Department of Behavioral Sciences, Karakoram International University Gilgit, Pakistan

**Keywords:** Anxiety, Depression, Gilgit-Baltistan, Pakistan, PHC Women

## Abstract

**Objective:**

To assess symptoms of anxiety and depression among women reporting to primary health care (PHC women) in Gilgit Baltistan (GB), Pakistan.

**Methods:**

This was a cross-sectional study conducted on PHC women belonging GB including other three provinces of Pakistan. PHQ-9 and GAD-7 were used to assess anxiety and depression. Descriptive and inferential statistical techniques were applied to analyze the collected data.

**Results:**

PHC women from GB reported higher level of depressive symptoms (*t*=7.58, *p*=0.00) and lower level of anxiety symptoms (*t*=8.3, *p*=0.00) when compared with cut-off score. Insignificant differences were found in depressive (*t*=1.5, *p*>.05) and anxiety (*t*=1.2, *p*>.05) scores between GB women and women from rest of Pakistan. However, inter-province differences in depressive (F=5.78, *p*= 00) and anxiety (F=4.5, *p*=0.00) symptoms were significant. Increasing age and lack of education were found significant risk factors for GB PHC women’s depressive and anxiety symptoms.

**Conclusions:**

PHC women from GB have higher level of depressive and lower level of anxiety symptoms like women from other provinces of Pakistan. Their demographics should be considered while addressing their emotional problems.

## INTRODUCTION

Globally, it is estimated that 4.4% of the population suffer from depressive disorders, and 3.6% from anxiety disorders.^1^ Worldwide, mental illnesses claim a major share in the years lived with disability (YLD) figures amounting for a good 32.4%.^2^

Among these illnesses, depression and related disorders remained the second highest cause on the list i.e. 9.6% of YLDs globally. Anxiety disorders were the seventh i.e. 3.5%.^3^ As per the available World Health Organization figures from Europe, 4 out of every 15 individuals had to face either of the two every year.^4^

Women remained the dominant gender in either disorder, with 5.1% women versus 3.6% men, and, 4.6% women versus 2.6% men in each respectively.^1^ Population estimates from Pakistan offered a prevalence of 4.2% for depressive and 3.5% for anxiety disorders.^1^ The women in Pakistan were found to be having two to three times more anxiety and depressive disorders than their male counterparts^5^, which is similar to the estimates from elsewhere.^6^

The landmark study in the northern Hindu Kush areas by Mumford et.al, although not precisely within the Gilgit Baltistan (GB) region of Pakistan, highlighted the significant prevalence of anxiety and depressive disorders, especially those in the women folk.^7^ In one of the recent most study from the country a 65% prevalence of depression was found in the women from a cosmopolitan city.^8^

The factors that had been highlighted in the national population associated with the two illness paradigms included increasing age, lesser education, abuse, and violence, being a housewife, relationship problems, being single, having more than four children and housing problems.^5^,^9^,^10^

The natives of GB have their ancestral roots in the region for thousands of years. This region was far away from the rest of the country/world and lied at the foot of some of the world’s largest mountain ranges, glaciers, and peaks. The medical facilities being scarce here and the region being less researched drew our attention towards the problem. We intended to find out the prevalence of anxiety and depression in the women of GB attending a primary health care (PHC) clinic.

## METHODS

This is a cross-sectional study that was conducted at the outpatient department, Combined Military Hospital Gilgit from January 2017 to June 2017. After approval of research protocols from institutional ethical review committee, data were collected from women presented for the treatment of different physical symptoms. Before collecting data verbal consent was also taken from individual research participants. The study enrolled 422 PHC women age ranged from 17 to 81 years. Three categories of women: severe morbidity, intellectual disability and other disability, which made them unable to answer question, were not included in the study. Administering PHQ-9 assessed participants’ depressive symptoms and GAD-7 was used to measure their anxiety symptoms.^11^,^12^ Demographic information form was administered to collect participants’ demographic information like age, presenting problems, living province, education level, occupation etc.

Collected data were analyzed using descriptive and inferential statistical techniques by SPSS (v.21). Based on assessment two different groups were defined as follows: Group-1 included those women who belong to GB and Group-2 comprised women from other provinces of Pakistan. Descriptive statistical techniques (mean & standard deviation) were used to summarize research data. For hypotheses testing inferential statistical techniques (one sample t-test, independent sample t-test, & ANOVA) were used. Level of significance *p*≤0.05 was considered significant and effect sizes of associations were also calculated (for t-test “*d*” and for ANOVA “ω^2^”).

## RESULTS

Out of the total participants (422); 309 (73%) were from GB and rest of them were from other provinces of Pakistan. Participants’ presenting problems were: 43(10.2%) hypertension, 15(3.6%) diabetes mellitus, 12(2.8%) ischemic heart disease, 9(2.1%) asthma, 8(1.9%) tuberculosis, 11(2.6%) hepatitis, 29(6.9%) psychiatric illnesses, and 295(69.9%) reported “other” medical problems. Most of the research participants were married (71.5%), majority of them were housewives (50.9%), and a good number of them (32.4%) had 14 years and above education level. Detail demographics are presented in Table-I.

**Table-I:** Demographic characteristics of research participants.

*S. No.*	*Demographics*	*Mean (SD) [Range]*	*n (%)*
1.	Age, in years	29.1(10.0) [17-81]	
2.	Living Province		
	Gilgit-Baltistan		309(73%)
	Punjab		58(14%)
	Sindh		39(9%)
	KPK		16(4%)
3.	Educational Level		
	Illiterate		48(11.3)
	Literate		77(18.2)
	10 years education		89(21.0)
	12 years education		71(16.6)
	14 years and above		137(32.4)
4.	Marital Status		
	Single		101(23.9)
	Married		302(71.5)
	Divorced		19(4.5)
5.	Occupation		
	Housewives		215(50.9)
	Government Employees		49(11.6)
	Students		108(25.6)
	Unemployed		24(5.6)
	Others		26(6.1)

In line with the recommendations of previous researches, the cut-off score of ≥6 for PHQ-9and ≥10 for GAD-7 was used to interpret data in this study.^13^,^14^

When compared with cut-off score, PHC women in GB reported higher level of depressive symptoms and lower level of anxiety symptoms with the small effect size (Table-II).^15^ There were no significant differences in depressive and anxiety symptoms between women from GB and those from the rest of Pakistan (Table-III).

**Table-II:** Comparison of participants’ anxiety and depressive symptoms against cut-off score.

*Scales*	*Cut-off score*	*N*	*M*	*SD*	*t*	*p*	*d*
PHQ-9	≥6	309	8.89	6.7	7.58	0.00	0.43
GAD-7	≥10	309	7.0	6.1	8.3	0.00	0.47

**Table-III:** Comparison of participants’ anxiety and depressive symptoms between GB and Pakistan.

	*Women from other Provinces*			*Women from GB*					

Scales	N	M	SD	N	M	SD	t	p	Cohen’s d
PHQ-9	113	7.74	7.4	309	8.89	6.7	1.5	.13	N/A
GAD-7	113	6.2	5.8	309	7.0	6.1	1.2	.2	N/A

However, ANOVA results indicated inter-province differences in depressive symptoms with a small effect size (Table-IV).^16^ Post-hoc (Hochberg) revealed that participants’ from KPK reported (M=13.63, SD =10.5) highest level of depressive symptoms as compared to participants from GB (M=8.89, SD=6.7, *p*=0.04), Punjab (M=6.19, SD=6.2, *p*=0.00), and Sindh (M=7.64, SD=6.58, *p*=0.01). PHC women from GB reported (M=8.89, SD=6.7) higher level of depressive symptoms as compared to PHC women from Punjab (M=6.19, SD=6.2, *p*=0.03). Participants’ living province also had significant effect on their reported level of anxiety symptoms with large effect size. Post-hoc indicated differences in anxiety symptoms only between participants from KPK (M=10.9, SD=8.0) and Punjab (M=5.0, SD=5.1, *p*=0.00).

**Table-IV:** Province-wise comparison of anxiety and depressive symptoms.

*Variable*	*Sources*	*df*	*SS*	*MS*	*F*	*ω^2^*
PHQ-9	Between Groups	3	802.7	267.5	5.7**	0.03
	Within Groups	418	19341.8	46.2		
	Total	421	20144.5			
GAD-7	Between Groups	3	496.7	165.5	4.5**	0.9
	Within Groups	418	15083.9	36.0		
	Total	421	15580.7			

** p<0.01

For women from GB, role of demographics in depressive and anxiety symptoms was also assessed. Participants’ age was positively and significantly correlated with depressive (*r*=0.14, *p*=0.01) and anxiety (*r*=0.17, *p*=0.00) symptoms indicated that when age increased depressive and anxiety symptoms also increased. There was a significant effect of level of education on depressive (F=5.86, *p*=0.00, ω^2^=0.05) symptoms with a small effect size and on anxiety symptoms with large effect size (F=8.5, *p*=0.00, ω^2^=0.8). Post-hoc analysis revealed that illiterate participants reported highest level of depressive (M=14.04, SD=6.6) and anxiety (Mean = 12.8, SD = 6.0) symptoms as compared to literate participants (Depression; M=9.19, SD=6.56, *p*=0.01, Anxiety; Mean=7.0, SD=5.6, *p*=0.00), participants with 10 years educations (Depression; M=9.25, SD=6.12, *p*=0.01, Anxiety; M=7.5, SD=6.6, *p*=0.00), participants with 12 years education (Depression; M=8.0, SD=6.8, *p*=0.00, Anxiety; M=6.0, SD=5.9, *p*=0.00), and participants with 14 and above year’s education (Depression; M=7.48, SD=6.4, *p*=0.00, Anxiety; M=5.7, SD=5.3, *p*=0.00). Participants’ marital status has not effect on their reported level of depressive (F=1.5, *p*=0.2) and anxiety (F=0.77, *p*=0.46) symptoms. There was insignificant effect of participants’ occupation on their reported level of depressive symptoms (F=2.09, *p*=0.08) but it has a significant effect on their anxiety symptoms with large effect size (F=3.5, *p*=0.00, ω^2^ = 0.9).Post-hoc indicated differences in anxiety symptoms only between housewives (M=7.6, SD=6.3) and unemployed participants (M=3.4, SD=4.0), *p*=0.04). The most occurring symptom of depression in GB was “*feeling tired or having little energy*” and the least occurring symptom was “*feeling bad about yourself or that you are a failure or have let yourself or your family down*” and the most occurring symptom of anxiety in GB was “*becoming easily annoyed or irritated*” and the least occurring symptoms was “*trouble relaxing*”.

## DISCUSSION

The current study shows that PHC women from GB reported higher level of depressive symptoms. However, they reported lower level of anxiety symptoms. Findings of current study are comparable with other studies reported from Pakistani context. Depression was the most common psychiatric disorder among married women visiting private family physicians.^9^ In another study 65% of married women visiting private hospitals, belonging to different socioeconomic classes were depressed irrespective of their socioeconomic groups.^8^ The scenario of GB is not much different as reported by Najam and Hussain; women in GB had lower level of mental health and higher level of psychological distress in terms of depression.^17^

Regarding prevalence of anxiety, the mean scores (M = 7.0) of the present study is slightly higher than past study reported from Pakistan (5.7) with similar sample, where 28% had borderline or pathological level of anxiety.^18^ In context of GB, 25% women visiting medical center suffered from anxiety.^19^ Najam and Hussain also found higher level of psychological distress in terms of anxiety among primary health care women in GB.^17^ However, other studies from Pakistan reported relatively higher level of anxiety symptoms among; women in a lower middle class semi-urban community, women with infertility, women with polycystic ovary syndrome, patients suffering from tuberculosis, and doctors serving in a tertiary care hospital.^20^-^24^

Taking into account the overwhelming prevalence of depression and anxiety among women in Pakistan, Zahidie and Jamali identified risk factors associated with depression among adult women within Pakistani geographical context are; marriage related problems, domestic violence, abuse either verbal or physical by in-laws, stressful life and poor social conditions, and pregnancy related concerns.^25^ Hussain et al. recognized following risk factors associated with depression in rural Pakistan: low educational status, having four or more children, being unmarried, being older, and living in a house where more than three people share single room.^10^ According to Mirza and Jenkins, being a female and housewife, middle age, low level of education, and financial and relationship problems were positively associated with anxiety and depressive disorders in Pakistan.^5^ Ali et al. reported age and lack of education as risk factors for depression and anxiety.^20^ Similarly, in the current study, age and education were found as significant risk factors associated with depression and anxiety for GB women. Additionally, GB women’s occupation was also proved to be a significant risk factor for their level of anxiety.

### Limitations

In the present study, small sample was included from Punjab, Sindh, and KPK while no case was included from Baluchistan. We did not include control group in our study.

## CONCLUSIONS

In conclusion, the present study shows that women coming to PHC in GB have higher level of depressive symptoms but their anxiety symptoms were lower when compared with cut-off score. Women’s age and level of education were found to be significant risk factors for their depressive and anxiety symptoms but their occupation tended to play an important role only in anxiety symptoms however. Based on findings, it is recommended to include mental health interventions as an integral part of the national health program with particular focus on women. Follow-up studies with more demographic and social controls are suggested for further clarification of the prevalence of depression and anxiety among women in GB.

### Author`s Contribution

**SH** conceived, designed and did statistical analysis & editing of manuscript.

**SA** did manuscript writing, review and final approval of manuscript.

**AZ & NJ** did data collection and manuscript writing.
